# Phenyl piperidine-1-carboxyl­ate

**DOI:** 10.1107/S1600536809050958

**Published:** 2009-12-04

**Authors:** Durre Shahwar, M. Nawaz Tahir, Naeem Ahmad, Saif Ullah, Muhammad Akmal Khan

**Affiliations:** a Department of Chemistry, Government College University, Lahore, Pakistan; b Department of Physics, University of Sargodha, Sargodha, Pakistan

## Abstract

In the title compound, C_12_H_15_NO_2_, the dihedral angle between the benzene ring and the basal plane of the piperidine ring (which is in a chair conformation) is 49.55 (8)°. In the crystal, mol­ecules are linked by C—H⋯O hydrogen bonds and very weak C–H⋯π inter­actions.

## Related literature

For related structures, see: Shahwar *et al.* (2009*a* ▶,*b* ▶).
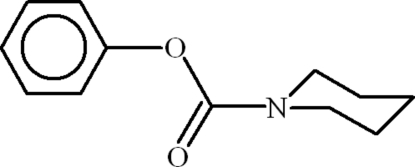


## Experimental

### 

#### Crystal data

C_12_H_15_NO_2_
*M* *_r_* = 205.25
Monoclinic, 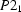

*a* = 6.2091 (2) Å
*b* = 7.6881 (3) Å
*c* = 11.2838 (4) Å
β = 97.211 (2)°
*V* = 534.39 (3) Å^3^
*Z* = 2
Mo *K*α radiation
μ = 0.09 mm^−1^
*T* = 296 K
0.28 × 0.11 × 0.09 mm

#### Data collection

Bruker Kappa APEXII CCD diffractometer
Absorption correction: multi-scan (*SADABS*; Bruker, 2005 ▶) *T*_min_ = 0.987, *T*_max_ = 0.993
6213 measured reflections
1422 independent reflections
1243 reflections with *I* > 2σ(*I*)
*R*_int_ = 0.022

#### Refinement

*R*[*F* ^2^ > 2σ(*F* ^2^)] = 0.035
*wR*(*F* ^2^) = 0.088
*S* = 1.05
1422 reflections
136 parameters
H-atom parameters constrained
Δρ_max_ = 0.14 e Å^−3^
Δρ_min_ = −0.22 e Å^−3^

### 

Data collection: *APEX2* (Bruker, 2007 ▶); cell refinement: *SAINT* (Bruker, 2007 ▶); data reduction: *SAINT*; program(s) used to solve structure: *SHELXS97* (Sheldrick, 2008 ▶); program(s) used to refine structure: *SHELXL97* (Sheldrick, 2008 ▶); molecular graphics: *ORTEP-3* (Farrugia, 1997 ▶) and *PLATON* (Spek, 2009 ▶); software used to prepare material for publication: *WinGX* (Farrugia, 1999 ▶) and *PLATON*.

## Supplementary Material

Crystal structure: contains datablocks global, I. DOI: 10.1107/S1600536809050958/hb5246sup1.cif

Structure factors: contains datablocks I. DOI: 10.1107/S1600536809050958/hb5246Isup2.hkl

Additional supplementary materials:  crystallographic information; 3D view; checkCIF report

## Figures and Tables

**Table 1:** Hydrogen-bond geometry (Å, °)

*D*—H⋯*A*	*D*—H	H⋯*A*	*D*⋯*A*	*D*—H⋯*A*
C2—H2⋯O2^i^	0.93	2.47	3.342 (2)	157
C5—H5⋯*Cg*2^ii^	0.93	2.99	3.632 (2)	128
C10—H10*A*⋯*Cg*2^iii^	0.97	2.97	3.848 (2)	151

Symmetry codes: (i) 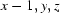; (ii) 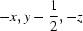; (iii) 
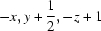
. *Cg*2 is the centroid of the C1–C6 ring.

**Fig. 1.**
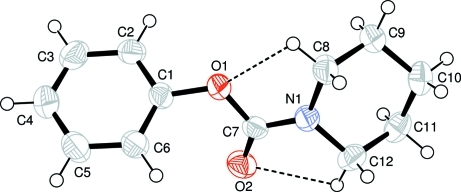
View of (I) with displacement ellipsoids drawn at the 50% probability level.

**Fig. 2.**
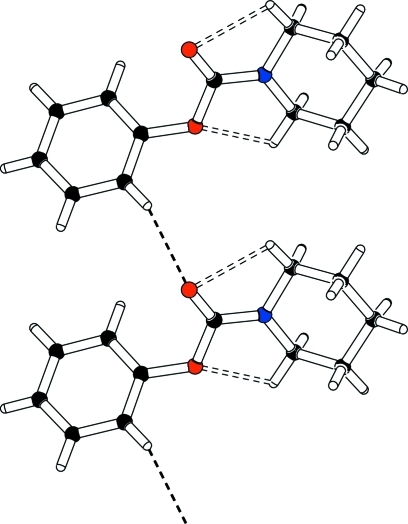
The partial packing of (I), which shows that molecules are linked in polymeric chains.

## supplementary crystallographic information

### Comment

We have recently reported the crystal structure of (II) Phenyl *N*-(2-methylphenyl)carbamate (Shahwar *et al.*, 2009*a*) and (III) Phenyl *N*-phenylcarbamate (Shahwar *et al.*, 2009*b*). The title compound (I, Fig. 1) is in continuation to synthesize various carbamates.

In (I), the benzene ring A (C1—C6) is of course planar. The group B (O1/O2/C7/N1/C8/C12) and C (C8/C9/C11/C12) are also planar with maximum r. m. s. deviations of 0.0127 and 0.0046 Å respectively, from the respective mean square planes. The dihedral angles between A/B, B/C and A/C are 56.37 (5)°, 50.95 (7)° and 49.55 (8)° respectively. The piperidine is in the chair conformation as the apical atoms N1 and C10 are at a distance of -0.6211 (26) and 0.6523 (30) Å respectively, from the basal plane (C8/C9/C11/C12). The molecules are stabilized in the form of polymeric chains (Table 1, Fig. 2). The C–H···π interactions (Table 1) also play a role in stabilizing the molecules.

### Experimental

Piperidine (0.01 *M*, 0.99 ml) and triethylamine (0.012 *M*, 1.66 ml) were added to 20 ml dichlorometnane in a 50 ml round bottom flask equipped with magnetic stirrer. Phenyl chloroformate (0.01 *M*, 1.26 ml) was added drop wise with continuous stirring of the contents of the flask. After complete addition the stirring was continued for 30 minutes. Extra dichloromethane was evaporated and then resulting solid was washed with 1*M* HCL and filtered to get pure product. Recrystallization of the crude product with ethyl acetate affoarded colourless needles of (I).

### Refinement

The H-atoms were positioned geometrically (C–H = 0.93–0.97 Å) and refined as riding with *U*_iso_(H) = 1.2*U*_eq_(C).

### Crystal data

**Table:**

C_12_H_15_NO_2_	*F*(000) = 220
*M**_r_* = 205.25	*D*_x_ = 1.276 Mg m^−^^3^
Monoclinic, *P*2_1_	Mo *K*α radiation, λ = 0.71073 Å
Hall symbol: P 2yb	Cell parameters from 1422 reflections
*a* = 6.2091 (2) Å	θ = 3.2–28.3°
*b* = 7.6881 (3) Å	µ = 0.09 mm^−^^1^
*c* = 11.2838 (4) Å	*T* = 296 K
β = 97.211 (2)°	Needles, colorless
*V* = 534.39 (3) Å^3^	0.28 × 0.11 × 0.09 mm
*Z* = 2	

### Data collection

**Table:**

Bruker Kappa APEXII CCD diffractometer	1422 independent reflections
Radiation source: fine-focus sealed tube	1243 reflections with *I* > 2σ(*I*)
graphite	*R*_int_ = 0.022
Detector resolution: 7.40 pixels mm^-1^	θ_max_ = 28.3°, θ_min_ = 3.2°
ω scans	*h* = −8→8
Absorption correction: multi-scan (*SADABS*; Bruker, 2005)	*k* = −9→10
*T*_min_ = 0.987, *T*_max_ = 0.993	*l* = −15→14
6213 measured reflections	

### Refinement

**Table:**

Refinement on *F*^2^	Primary atom site location: structure-invariant direct methods
Least-squares matrix: full	Secondary atom site location: difference Fourier map
*R*[*F*^2^ > 2σ(*F*^2^)] = 0.035	Hydrogen site location: inferred from neighbouring sites
*wR*(*F*^2^) = 0.088	H-atom parameters constrained
*S* = 1.05	*w* = 1/[σ^2^(*F*_o_^2^) + (0.0453*P*)^2^ + 0.0354*P*] where *P* = (*F*_o_^2^ + 2*F*_c_^2^)/3
1422 reflections	(Δ/σ)_max_ < 0.001
136 parameters	Δρ_max_ = 0.14 e Å^−^^3^
0 restraints	Δρ_min_ = −0.22 e Å^−^^3^

### Special details

**Table:**

Geometry. Bond distances, angles *etc*. have been calculated using the rounded fractional coordinates. All su's are estimated from the variances of the (full) variance-covariance matrix. The cell e.s.d.'s are taken into account in the estimation of distances, angles and torsion angles
Refinement. Refinement of *F*^2^ against ALL reflections. The weighted *R*-factor *wR* and goodness of fit *S* are based on *F*^2^, conventional *R*-factors *R* are based on *F*, with *F* set to zero for negative *F*^2^. The threshold expression of *F*^2^ > σ(*F*^2^) is used only for calculating *R*-factors(gt) *etc*. and is not relevant to the choice of reflections for refinement. *R*-factors based on *F*^2^ are statistically about twice as large as those based on *F*, and *R*- factors based on ALL data will be even larger.

### Fractional atomic coordinates and isotropic or equivalent isotropic displacement parameters (Å^2^)

**Table:**

	*x*	*y*	*z*	*U*_iso_*/*U*_eq_	
O1	−0.00238 (19)	0.6920 (2)	0.32910 (10)	0.0497 (4)	
O2	0.3128 (2)	0.83567 (19)	0.31583 (11)	0.0534 (4)	
N1	0.2483 (2)	0.7051 (2)	0.48833 (12)	0.0442 (4)	
C1	−0.0639 (3)	0.6857 (2)	0.20515 (14)	0.0395 (5)	
C2	−0.2721 (3)	0.7369 (3)	0.16604 (16)	0.0465 (6)	
C3	−0.3512 (3)	0.7125 (4)	0.04706 (18)	0.0599 (7)	
C4	−0.2238 (4)	0.6384 (3)	−0.03033 (18)	0.0631 (8)	
C5	−0.0147 (4)	0.5895 (3)	0.01023 (19)	0.0578 (7)	
C6	0.0681 (3)	0.6137 (3)	0.12898 (16)	0.0485 (6)	
C7	0.2001 (3)	0.7508 (2)	0.37356 (14)	0.0384 (5)	
C8	0.1076 (3)	0.6065 (3)	0.55852 (15)	0.0474 (6)	
C9	0.0725 (3)	0.7057 (3)	0.67042 (15)	0.0487 (6)	
C10	0.2875 (3)	0.7539 (3)	0.74199 (17)	0.0538 (6)	
C11	0.4301 (3)	0.8523 (3)	0.66545 (17)	0.0521 (6)	
C12	0.4601 (3)	0.7516 (3)	0.55382 (17)	0.0486 (5)	
H2	−0.35844	0.78703	0.21843	0.0558*	
H3	−0.49220	0.74669	0.01916	0.0719*	
H4	−0.27888	0.62132	−0.10998	0.0757*	
H5	0.07179	0.53993	−0.04228	0.0693*	
H6	0.21007	0.58190	0.15663	0.0582*	
H8A	0.17346	0.49472	0.58031	0.0569*	
H8B	−0.03120	0.58559	0.51088	0.0569*	
H9A	−0.01098	0.63469	0.71919	0.0584*	
H9B	−0.00986	0.81054	0.64855	0.0584*	
H10A	0.26113	0.82542	0.80952	0.0646*	
H10B	0.36158	0.64910	0.77265	0.0646*	
H11A	0.36452	0.96411	0.64339	0.0626*	
H11B	0.57069	0.87341	0.71114	0.0626*	
H12A	0.54102	0.82153	0.50311	0.0584*	
H12B	0.54274	0.64671	0.57525	0.0584*	

### Atomic displacement parameters (Å^2^)

**Table:**

	*U*^11^	*U*^22^	*U*^33^	*U*^12^	*U*^13^	*U*^23^
O1	0.0416 (7)	0.0749 (9)	0.0328 (6)	−0.0111 (7)	0.0056 (5)	0.0005 (7)
O2	0.0542 (7)	0.0587 (8)	0.0497 (7)	−0.0108 (7)	0.0159 (6)	0.0040 (7)
N1	0.0396 (7)	0.0565 (9)	0.0366 (7)	−0.0128 (7)	0.0047 (6)	−0.0020 (7)
C1	0.0429 (9)	0.0422 (9)	0.0336 (8)	−0.0044 (8)	0.0057 (7)	0.0045 (8)
C2	0.0404 (9)	0.0524 (10)	0.0481 (10)	−0.0005 (9)	0.0106 (8)	0.0029 (9)
C3	0.0429 (10)	0.0806 (16)	0.0539 (11)	0.0016 (10)	−0.0034 (8)	0.0127 (12)
C4	0.0619 (13)	0.0892 (18)	0.0366 (10)	−0.0035 (12)	−0.0003 (9)	0.0017 (10)
C5	0.0645 (12)	0.0676 (13)	0.0425 (10)	0.0097 (10)	0.0120 (9)	−0.0034 (9)
C6	0.0469 (10)	0.0573 (11)	0.0415 (9)	0.0120 (9)	0.0068 (8)	0.0056 (9)
C7	0.0368 (8)	0.0406 (8)	0.0395 (8)	−0.0010 (8)	0.0110 (7)	−0.0063 (8)
C8	0.0497 (10)	0.0552 (10)	0.0366 (9)	−0.0162 (9)	0.0027 (7)	0.0026 (8)
C9	0.0488 (9)	0.0599 (12)	0.0376 (9)	−0.0028 (9)	0.0066 (7)	0.0040 (8)
C10	0.0637 (12)	0.0561 (11)	0.0386 (9)	−0.0001 (10)	−0.0054 (8)	−0.0060 (9)
C11	0.0461 (10)	0.0503 (11)	0.0559 (11)	−0.0050 (9)	−0.0096 (8)	−0.0079 (9)
C12	0.0360 (8)	0.0536 (10)	0.0549 (10)	−0.0043 (8)	0.0002 (7)	−0.0031 (9)

### Geometric parameters (Å, °)

**Table:**

O1—C1	1.4037 (19)	C2—H2	0.9300
O1—C7	1.371 (2)	C3—H3	0.9300
O2—C7	1.206 (2)	C4—H4	0.9300
N1—C7	1.339 (2)	C5—H5	0.9300
N1—C8	1.463 (2)	C6—H6	0.9300
N1—C12	1.470 (2)	C8—H8A	0.9700
C1—C2	1.370 (3)	C8—H8B	0.9700
C1—C6	1.376 (3)	C9—H9A	0.9700
C2—C3	1.383 (3)	C9—H9B	0.9700
C3—C4	1.373 (3)	C10—H10A	0.9700
C4—C5	1.374 (3)	C10—H10B	0.9700
C5—C6	1.386 (3)	C11—H11A	0.9700
C8—C9	1.514 (3)	C11—H11B	0.9700
C9—C10	1.517 (3)	C12—H12A	0.9700
C10—C11	1.515 (3)	C12—H12B	0.9700
C11—C12	1.510 (3)		
			
C1—O1—C7	119.90 (13)	C1—C6—H6	121.00
C7—N1—C8	125.71 (14)	C5—C6—H6	121.00
C7—N1—C12	119.99 (15)	N1—C8—H8A	110.00
C8—N1—C12	114.30 (14)	N1—C8—H8B	110.00
O1—C1—C2	116.02 (15)	C9—C8—H8A	110.00
O1—C1—C6	121.82 (16)	C9—C8—H8B	110.00
C2—C1—C6	121.75 (16)	H8A—C8—H8B	108.00
C1—C2—C3	118.64 (18)	C8—C9—H9A	109.00
C2—C3—C4	120.71 (19)	C8—C9—H9B	109.00
C3—C4—C5	119.83 (19)	C10—C9—H9A	109.00
C4—C5—C6	120.4 (2)	C10—C9—H9B	109.00
C1—C6—C5	118.69 (18)	H9A—C9—H9B	108.00
O1—C7—O2	123.27 (15)	C9—C10—H10A	109.00
O1—C7—N1	110.54 (14)	C9—C10—H10B	109.00
O2—C7—N1	126.16 (16)	C11—C10—H10A	109.00
N1—C8—C9	110.42 (17)	C11—C10—H10B	109.00
C8—C9—C10	110.97 (16)	H10A—C10—H10B	108.00
C9—C10—C11	110.91 (16)	C10—C11—H11A	109.00
C10—C11—C12	111.21 (18)	C10—C11—H11B	109.00
N1—C12—C11	110.36 (15)	C12—C11—H11A	109.00
C1—C2—H2	121.00	C12—C11—H11B	109.00
C3—C2—H2	121.00	H11A—C11—H11B	108.00
C2—C3—H3	120.00	N1—C12—H12A	110.00
C4—C3—H3	120.00	N1—C12—H12B	110.00
C3—C4—H4	120.00	C11—C12—H12A	110.00
C5—C4—H4	120.00	C11—C12—H12B	110.00
C4—C5—H5	120.00	H12A—C12—H12B	108.00
C6—C5—H5	120.00		
			
C7—O1—C1—C2	−139.17 (18)	O1—C1—C2—C3	−171.8 (2)
C7—O1—C1—C6	48.0 (2)	C6—C1—C2—C3	1.0 (3)
C1—O1—C7—O2	18.0 (3)	O1—C1—C6—C5	171.01 (18)
C1—O1—C7—N1	−163.81 (15)	C2—C1—C6—C5	−1.4 (3)
C8—N1—C7—O1	−0.1 (2)	C1—C2—C3—C4	0.0 (4)
C8—N1—C7—O2	178.06 (18)	C2—C3—C4—C5	−0.7 (4)
C12—N1—C7—O1	178.78 (15)	C3—C4—C5—C6	0.3 (4)
C12—N1—C7—O2	−3.1 (3)	C4—C5—C6—C1	0.7 (3)
C7—N1—C8—C9	−124.65 (18)	N1—C8—C9—C10	−54.5 (2)
C12—N1—C8—C9	56.5 (2)	C8—C9—C10—C11	54.4 (2)
C7—N1—C12—C11	124.72 (18)	C9—C10—C11—C12	−54.5 (2)
C8—N1—C12—C11	−56.3 (2)	C10—C11—C12—N1	54.2 (2)

### Hydrogen-bond geometry (Å, °)

**Table:**

*D*—H···*A*	*D*—H	H···*A*	*D*···*A*	*D*—H···*A*
C2—H2···O2^i^	0.93	2.47	3.342 (2)	157
C5—H5···Cg2^ii^	0.93	2.99	3.632 (2)	128
C10—H10A···Cg2^iii^	0.97	2.97	3.848 (2)	151

Symmetry codes: (i) *x*−1, *y*, *z*; (ii) −*x*, *y*−1/2, −*z*; (iii) −*x*, *y*+1/2, −*z*+1.
